# Lumateperone Normalizes Pathological Levels of Acute Inflammation through Important Pathways Known to Be Involved in Mood Regulation

**DOI:** 10.1523/JNEUROSCI.0984-22.2022

**Published:** 2023-02-01

**Authors:** Sophie Dutheil, Luke S. Watson, Robert E. Davis, Gretchen L. Snyder

**Affiliations:** Intra-Cellular Therapies, Inc., Alexandria Center for Life Sciences, New York, New York 10016

**Keywords:** anti-inflammatory, blood–brain barrier, depression, immune system, lumateperone, stress

## Abstract

Lumateperone is indicated for the treatment of schizophrenia in adults and for depressive episodes associated with bipolar I or II disorder (bipolar depression) in adults, as monotherapy and as adjunctive therapy with lithium or valproate (Calabrese et al., 2021). It is currently under evaluation for the treatment of major depressive disorder (www.ClinicalTrials.gov). Lumateperone acts by selectively modulating serotonin, dopamine, and glutamate neurotransmission in the brain. However, other mechanisms could be involved in the actions of lumateperone, and because of the connection between the immune system and psychiatric health, we hypothesized that lumateperone might improve symptoms of depression, at least in part, by normalizing pathologic inflammation. Here, we show that in male and female C57BL/6 mice subjected to an acute immune challenge, lumateperone reduced aberrantly elevated levels of key proinflammatory cytokines (e.g., IL-1β, IL-6, and TNF-α) in both brain and serum; lumateperone also reduced proinflammatory cytokines in male mice under acute behavioral stress. Further, we demonstrate that lumateperone altered key genes/pathways involved in maintaining tissue integrity and supporting blood–brain barrier function, such as claudin-5 and intercellular adhesion molecule 1. In addition, in acutely stressed male Sprague Dawley rats, lumateperone conferred anxiolytic- and antianhedonic-like properties while enhancing activity in the mammalian target of rapamycin complex 1 pathway in the PFC. Together, our preclinical findings indicate that lumateperone, in addition to its ability to modulate multiple neurotransmitter systems, could also act by reducing the impact of acute inflammatory challenges.

**SIGNIFICANCE STATEMENT** Lumateperone is indicated in adults to treat schizophrenia and depressive episodes associated with bipolar I or II disorder, as monotherapy and adjunctive therapy with lithium or valproate. Because aberrant immune system activity is associated with increased depressive symptoms, the relationship between lumateperone and immune function was studied. Here, lumateperone reduced the levels of proinflammatory cytokines that were increased following an immune challenge or stress in mice. Additionally, lumateperone altered genes and pathways that maintain blood–brain barrier integrity, restored an index of blood–brain barrier function, reduced anxiety-like behavior in rodents, and enhanced mammalian target of rapamycin complex 1 pathway signaling in the PFC. These results highlight the anti-inflammatory actions of lumateperone and describe how lumateperone may reduce immune pathophysiology, which is associated with depressive symptoms.

## Introduction

Lumateperone is indicated for the treatment of schizophrenia in adults and for depressive episodes associated with bipolar I or II disorder (bipolar depression) in adults, as monotherapy and as adjunctive therapy with lithium or valproate. It is currently under evaluation for the treatment of major depressive disorder (MDD; www.ClinicalTrials.gov). Lumateperone simultaneously modulates serotonin, dopamine, and glutamate levels in the brain, all of which are key neurotransmitters implicated in serious mental illness (P. Li et al., 2014; Snyder et al., 2015; Intra-Cellular Therapies, Inc., 2021). Specifically, lumateperone directly interacts with serotonin 5-HT_2A_, dopamine D_2_ and D_1_ receptors, and the serotonin reuptake transporter (Snyder et al., 2015). Lumateperone also indirectly enhances NMDA- and AMPA-mediated neurotransmission and prefrontal glutamatergic receptor-mediated neurotransmission (Titulaer et al., 2022). The pharmacology of this agent is consistent with the potential to treat symptoms of depression. It was shown to improve depressive symptoms in adult patients with schizophrenia with comorbid depression (Lieberman et al., 2016) and when given as monotherapy or adjunctive therapy to treatment with lithium or valproate in adult patients with bipolar I or bipolar II disorder who were experiencing major depressive episodes (Calabrese et al., 2021; Intra-Cellular Therapies, Inc., 2021). Antidepressant-like activity of lumateperone has also been demonstrated in a mouse model of chronic social defeat (Snyder et al., 2015).

Despite half a century of research, the pathophysiology of depressive and mood disorders remains largely elusive. Immunologic disturbances have been reported in subsets of patients suffering from generalized anxiety, MDD, and/or schizophrenia (Mechawar and Savitz, 2016; Zhang et al., 2016; Herman and Pasinetti, 2018; Beurel et al., 2020). In addition, acute exposure to infectious agents and subsequent heightened immune activity can also lead to transient depressive symptoms (e.g., anhedonia, fatigue, lethargy, and depressed mood) (Nazimek et al., 2017; Beurel et al., 2020). This concept is supported by studies in which direct administration of pro-inflammatory factors induced stress-like and/or depressive-like effects in patients or animals (Mechawar and Savitz, 2016) and studies showing that adverse symptoms were reversed by direct blockade of certain immune pathways, such as interleukin (IL)-1β (Koo and Duman, 2008, 2009). Here, we explored the complex interactions between lumateperone and relevant behavioral and biological immune/inflammatory pathways that could contribute to its ability to improve symptoms of depression, especially in subjects exhibiting heightened immune responses. Our results show that, in mice subjected to an acute immune challenge or acute behavioral stress, lumateperone reduced aberrantly elevated levels of proinflammatory cytokines (e.g., IL-1β, IL-6, and TNF-α) in both brain and serum. We also examined potential effects of lumateperone on microglia enriched from rat hippocampus, providing a greater understanding of the mechanism by which this drug modulates neuroinflammation. Further, we demonstrate that lumateperone altered key genes/pathways involved in tissue integrity and maintenance of blood–brain barrier (BBB), such as claudin-5 (Cldn5) and intercellular adhesion molecule 1 (Icam1), conferred antianhedonic properties, and reinforced BBB protection during inflammatory and stress challenge. We also show that acute injections of lumateperone stimulated activity in the mammalian target of rapamycin complex 1 (mTORC1) pathway in the PFC and reduced anxiety in naive rats. Together, we highlight preclinical findings that provide new mechanisms which may contribute to lumateperone's ability to modulate certain mood states.

## Materials and Methods

### Animals

Adult, male or female C57BL/6 mice (The Jackson Laboratory) weighing 28–30 or 20–25 g, respectively, at the time of the experiment were housed in groups of 4 or 5 in small cages. Adult, male Sprague Dawley rats (Charles River Laboratories) weighing 175–200 g at the time of arrival after shipping were housed in pairs. All animals were housed under standard laboratory housing conditions with a 12 h light/dark cycle and *ad libitum* access to food and water. Animal use and procedures were in accordance with the National Institutes of Health guidelines and approved by Institutional Animal Care and Use Committee guidelines established by Mispro Biotech Services.

### Drugs and experimental design

Lumateperone, also known as ITI-007 or IC200056 tosylate salt (P. Li et al., 2016), was provided by the Medicinal Chemistry Department at Intra-Cellular Therapies, Inc. All other reagents were obtained in the highest purity available from Sigma-Aldrich unless otherwise noted. For most experiments, mice or rats at least 8 weeks of age received an intraperitoneal (i.p.) injection of lumateperone (0.3, 1, 3, or 8 mg/kg) or its vehicle (v/v: 5% DMSO, 5% Tween 20, 15% polyethylene glycol [PEG] 400, and 75% pure HPLC water). Some rodents were given a cotreatment with a subcutaneous (s.c.) injection of lipopolysaccharide (LPS, 500 µg/kg; Sigma-Aldrich, reference #0127:B8) diluted in 0.9% injectable saline while control group animals received injections of all vehicles matching the experimental conditions. In experiments in which delayed administration of lumateperone was studied, mice (*n* = 4–9 per group) first received a s.c. injection of LPS or saline followed by an i.p. injection of lumateperone (3 mg/kg) or its vehicle 30 min later. In experiments using restraint stress, mice assigned to the restraint stress group received a single injection of lumateperone (3 mg/kg) or its vehicle and were immediately placed in a rodent restraint bag. In behavioral experiments, rats received a pretreatment of lumateperone (1 mg/kg) or its vehicle on day 1. On day 2, either no injection for naive rats, or saline or LPS was injected (1 mg/kg, s.c.). On day 3, rats received another injection of either lumateperone or saline and were tested the following day (day 4).

### Tissue collection

Mice were killed 2 h after lumateperone injection (for cotreatment studies with LPS) or application of restraint stress for sample collection. Rats were killed 18 h after LPS injection for sample collection. Hippocampi from mice and rats were rapidly dissected under RNase-free conditions, and placed in 1.5 ml Eppendorf tubes. When appropriate, samples were snap frozen in liquid nitrogen before storage at −80°C until further analyses. Trunk blood was collected from mice into serum collection tubes (BD Microtainer Blood Collection Tubes), allowed to clot at room temperature for 1 h, then centrifuged at 1500 × *g* for 10 min at 4°C.

### Multiplex assays

In mouse serum, protein levels of IL-1β, IL-6, IL-10, and TNF-α were measured using a V-Plex Meso Scale Discovery (MSD) Multiplex spot assay Mouse Neuroinflammation 1 panel (Meso Scale Diagnostics). All samples were run in duplicates or triplicates according to manufacturer instructions and analyzed with MSD Discovery Workbench software (Meso Scale Diagnostics).

### qRT-PCR

Mouse hippocampal tissue was homogenized with glass beads in 1 ml of TRIzol reagent using a BeadBeater (Biospec Products). Heavy phase-lock gel tubes (QuantaBio; VWR International) enabled separation of phases following the addition of 400 µl chloroform to the sample and centrifugation at 12,000 rpm for 10 min at room temperature. RNA was extracted using RNeasy kit (QIAGEN). For cDNA synthesis, 2 µg of total RNA was used (SuperScript IV Reverse Transcriptase; Fisher Scientific). The purity and concentration of RNA were measured with a Nanodrop spectrophotometer; the optical density (OD) 260/280 and OD 260/230 were within 1.8–2.3. In the hippocampus, four key markers of pro- and anti-inflammatory cytokines and chemokines (*Il1b*: ID Mm00434228_m1, *Tnfa*: ID Mm00443258_m1, *Il6*: ID Mm00446190_m1, and *Il10*: ID Mm01288386_m1; Fisher Scientific) were initially chosen for analysis (*n* = 5–12 per group). In subsequent experiments, the transcripts for other markers of inflammation were chosen for analysis, including *Icam*1 (ID Mm00516023_m1; a cell adhesion molecule involved in immune cell migration), *Cldn5* (ID Mm00727012_s1; a tight junctions protein), colony stimulating factor 1 (*Csf1*: ID Mm00432686_m1; a factor that regulates microglia function) and its receptor *Csf1r* (ID Mm01266652_m1), and the nucleotide binding and oligomerization domain-like receptor family pyrin domain-containing three inflammasome complex (*Nlrp3*: Mm00840904_m1). *Gapdh* (ID Mm99999915_g1) was chosen as a housekeeping gene. QuantStudio 7 (Fisher Scientific) was used for analyzing the plates (MicroAmp Optical 384-well plates; Applied Biosystems, and Fisher Scientific) that were loaded with TaqMan Universal Master Mix II without uracil-DNA glycosylate in a 20 µl reaction volume using 100 ng cDNA per well. All mRNAs were measured by qRT-PCR on ABI Prism 7900HT system using TaqMan Gene Expression Assays. Ct values of genes of interest were normalized to that of the reference gene (*Gapdh*).

### NanoString

The mouse neuropathology panel included 770 genes associated with themes of neurotransmission, neuron–glia interaction, neuroplasticity, cell structure integrity, neuroinflammation, and metabolism. A total of 13 housekeeping genes were used for expression normalization (*Aars:* NM_146217.4, *Asb10*: NM_080444.4, *Ccdc127*: NM_024201.3, *Cnot10*: NM_153585.5, *Csnk2a2*: NM_009974.3, *Fam104a*: NM_138598.5, *Gusb*: NM_010368.1, *Lars*: NM_134137.2, *Mto1*: NM_026658.2, *Supt7l*: NM_028150.1, *Tada2b*: NM_001170454.1:3224, *Tbp*: NM_013684.3:70, and *Xpnpep1*: NM_133216.3:1826, see Extended Data Fig. 1-1). Hippocampal RNA was extracted using QIAGEN microkit and was evaluated by the Agilent 2100 Bioanalyzer (Agilent Technologies) to assess RNA concentration, quality, and integrity. Sample preparation, hybridization, and detection (100 ng per sample, *n* = 5 or 6 per group) were conducted at the New York University School of Medicine Langone Health Genome Technology Center (RRID: SCR_017929) according to the manufacturer's instructions (NanoString Technologies). The normalized data were transformed to log2 score to express the fold change. NanoString results (raw and normalized counts) were derived from RCC files using the nSolver software (version 2.6; NanoString Technologies). We also used a complementary gene software analysis, ROSALIND Advanced Analysis Software (NanoString Technologies) that provides comprehensive free cloud-based data analysis for nCounter data by directly analyzing raw RCC files generated from NanoString. Data were imported into ROSALIND Advanced Analysis Software for normalization, calculation of fold changes, *p* values, identification of enriched pathways, and heatmaps.

### Microglia enrichment

The microglia enrichment protocol was based on discussions and protocol from Dr. Helene Hirbec (Institut de Génomique Fonctionnelle, Université de Montpellier Toulouse). In male adult rats (8-9 weeks old, *n* = 8–12 per group) that were previously treated with either LPS and lumateperone or vehicle 1 d prior (18 h), hippocampi were dissected and placed in 1 ml of medium A solution containing 0.6% glucose and 15 mm HEPES. Brain tissue was then processed through a Dounce homogenizer followed by passages through 16- and 20-gauge needles; 1 ml of medium A was added to wash cell suspension, which was passed through a 70 mm cell strainer (Fisher Scientific; reference #22-363-548) and remained on ice. Next, 6 ml of 100% Percoll solution (9 parts of Percoll [GE Healthcare] and 1 part of HBSS) was added to obtain a 75% Percoll solution. The 75% Percoll cell suspension was then underlaid a layer of 25% Percoll solution containing Phenol red, which had a layer of PBS on top. The discontinuous Percoll density gradient was layered as follows: 75%, 25%, and 0% isotonic Percoll (PBS) to isolate hippocampal microglia. The gradients were then centrifuged at 4°C for 25 min at 3000 rpm in swinging buckets with minimal acceleration and deceleration and no brake. After centrifugation, the top layer containing myelin and debris (interface PBS/25% Percoll) was removed and the cellular layer at the 25%/75% interphase was collected and washed. Pilot experiments compared the gene expression of the different fractions and validated the presence of microglia in the interphase layer. The final pellet was resuspended in 350 µl of Buffer RLT from QIAGEN microkit to perform RNA extraction according to the manufacturer's instructions.

### Restraint stress protocol

Acute restraint stress was performed using special rodent decapicone restraint bags in the traditional triangle shape (Braintree Scientific; reference #MDC-200). Mice (*n* = 11–13 per group) were maintained in their restraint bag while placed on a secure surface at room temperature for 2 h. Mice were killed at the end of the 2 h stress session, and whole hippocampal samples were collected. Control, nonstressed mice remained in their home cages in an adjoining room and were killed for sample collection at the same time point as stressed mice.

### BBB permeability assay

Sodium fluorescein (NaFl) permeability assay was performed as previously described (Olsen et al., 2007) with minor adjustments. Mice (*n* = 4–8 per group) were administered lumateperone and either dosed with LPS or restrained as described above. At 45 min before tissue collection, mice received 200 µl of 10% NaFl (catalog #F6377, MilliporeSigma), i.p. Mice were then killed via isoflurane overdose, and blood was collected via cardiac stick and allowed to clot while protected from light. Mice were perfused with 15 ml 1 × PBS solution. Brains were then excised and flash frozen, protected from light. Serum was collected from blood samples via centrifugation at 1500 × *g* at 4°C for 10 min. Brains were homogenized in 1 × PBS and centrifuged at 10,000 × *g* at 4°C for 10 min, and the supernatant was collected for both protein concentration via Pierce BCA Protein Assay and further analysis. Proteins from both serum and tissue homogenate were extracted via trichloroacetic acid precipitation (catalog #T6399, MilliporeSigma) on ice, and centrifuged at 10,000 × *g* at 4°C for 10 min. Samples were run in duplicate on a FITC Filter spectrophotometer (EnVision 2105, PerkinElmer; excitation: 485 nm, emission: 535 nm). The average fluorescence of sham mice was subtracted from each value before calculation. Tissue homogenate fluorescent readings were first normalized to total protein concentration, and the cerebrum/serum ratio of arbitrary fluorescent units was calculated.

### Western blotting

Prefrontal cortical tissue was dissected from the rat brain and was immediately homogenized in Buffer A containing 0.32 m sucrose, 20 mm HEPES, pH 7.4, 1 mm EDTA, 1 × protease inhibitor and phosphatase inhibitor cocktails 2 and 3 (P0044 and P5726, Sigma-Aldrich). Synaptoneurosome-enriched fractions were prepared and sonicated in RIPA buffer. Protein concentration was determined by BCA protein assay (Pierce Biotechnology). Equal amounts of protein were loaded and separated on an SDS-PAGE gel (Bio-Rad, Criterion Tris-HCl Protein Gel). After electrophoresis, the proteins were transferred to nitrocellulose membranes and after blocking in TBS-Licor Buffer (Licor, Intercept [TBS] Blocking Buffer) blots were incubated in the appropriate primary antibody for phosphorylated (phosphor)-Akt thr308, phosphor-extracellular signal-regulated kinase (ERK), phosphor-mTOR (ser2448), phospho-P70S6K (Thr389), and their respective total protein levels, and β-actin (see references and dilutions in Table 1) in 1× TBS Licor Buffer + 0.1% Tween-20. Membranes were developed using Licor Odyssey system. The intensity of the protein bands was quantified using image analysis software (Image Studio, Lite, version 5.2). For each blot, the adjacent background signal was subtracted to the median background value surrounding each band from the target band. Resultant values were normalized to the average signal for the total (nonphosphorylated) protein levels to simplify gel analysis and reduce inter- and intra-gel variability. Data are expressed in percentages as change versus control levels.

**Table 1.** Western blot antibodies references and concentrations

kDa	Antibody name	Source	Brand	Reference	Dilution
289	Phospho-mTOR (Ser2448)	Rabbit	Cell Signaling	5536	1:1000
289	mTOR	Mouse	Cell Signaling	4517	1:1500
70-85	Phospho-p70 S6 kinase (Thr389)	Mouse	Cell Signaling	9206	1:600
70-85	p70 S6 kinase	Rabbit	Cell Signaling	2708	1:1000
60	Phospho-Akt (Thr308)	Rabbit	Cell Signaling	4056	1:400
60	Akt (pan)	Mouse	Cell Signaling	2920	1:700
45	β-actin	Mouse	Cell Signaling	3700	1:6000
44-42	Phospho-p44/42 MAPK (Erk1/2)	Rabbit	Cell Signaling	4370	1:1500
44-42	p44/42 MAPK (Erk1/2)	Mouse	Cell Signaling	4696	1:1500

MAPK, mitogen-activated protein kinase; mTOR, mammalian target of rapamycin.

### Behavioral evaluation

All behavioral tests were performed in the morning with adult male Sprague Dawley rats (*n* = 9–11 per group for the experiments with LPS, and *n* = 13 or 14 per group for the naive rats, Charles River Laboratories).

#### Novelty suppressed feeding test (NSFT)

This test measures consumption of a familiar food in a novel environment, relying on rodents' aversion to eating in a novel environment after a period of food deprivation (Ramaker and Dulawa, 2017). Rats were food-deprived overnight and placed in an open field (76.5 × 76.5 × 40 cm^3^) with a small amount of food pellets (6 pellets total). At the time of the test, rats were exposed to the open field for the first time (novelty) and allowed to explore it for a maximum of 15 min under red light. The latency for the animal to approach the food pellets and take its first bite was manually scored. A home cage feeding test (HCFT) was performed afterward to ensure latency measured in NSFT was not a matter of difference in hunger. A home cage food intake analysis evaluated the amount of food eaten (in grams) over a period of 10 min following the end of the entire test session.

#### Novelty induced hypophagia (NIH)

This conflict-based behavioral task assesses the impact of environmental stressors on conditioned approach response for a palatable food reward (Ramaker and Dulawa, 2017). Rats were habituated with diluted (1:3 milk/water) sweetened condensed milk (Nestle) which was accessible in their home cage for 1 h each day for 3 consecutive days. Initially, animals were tested in their home cage under normal lighting. For testing after drug treatment, the latency to drink was recorded after rats were placed in a novel clean cage of the same dimensions with no bedding and under dim lighting (∼50 lux) with white paper under the cages to enhance aversion.

#### Open field test (OFT)

Rats were placed in an open field box (76.5 × 76.5 × 40 cm^3^) under dim lighting, and locomotor activity over a 10 min period was measured using ANY-Maze Software (Stoelting).

#### Reward sniffing test

Reward sniffing test is also known as female urine sniffing test. In this anhedonia-based assay, rats were brought to a well-ventilated testing room under dim lighting. A sterile cotton-tipped applicator was attached to one wall in the home cage for 1 h to habituate rats to this new object. For the two phases of the 5 min test, rats were first exposed to a new cotton tip dipped in sterile water as a control that was removed at the end of the 5 min; 45 min later, another cotton tip previously dipped into fresh rat urine collected from females of the same strain was attached to the cage wall. Male behavior was video recorded and latency to first sniff of the cotton tip and total time spent sniffing the cotton-tipped applicator were determined.

### Statistical analysis

Data are expressed as mean ± SEM. All statistical analyses were performed using GraphPad version 9 or earlier (GraphPad Software). Sample sizes for the experiments were calculated using expected effect size and variance based on previous data. The Kolmogorov–Smirnov test was used as a test of normality. Unpaired *t* tests two-sided were used for comparison between two groups. When the normal distribution was not confirmed, the Mann–Whitney *U* test was used to compare the mean ranks of two groups. Multiple group comparisons were made using one-way ANOVA followed by a Bonferroni *post hoc* test or Tukey's multiple comparison test. NanoString nCounter analysis was based on multivariate linear regression with Benjamani–Yekutieli adjustment. Probability value was noted in each figure, and details on specific tests used are stated in the figure legends. Outliers were removed using the median absolute deviation equation (median ± 2.5 times the median absolute deviation method for outlier detection), a more robust measure of dispersion than the mean ± 2 or 3 SDs (Leys et al., 2013). Data from this study are available from the corresponding author (S.D.), on reasonable request.

## Results

### Lumateperone dose-dependently normalized pro-inflammatory state

**Figure 1.**
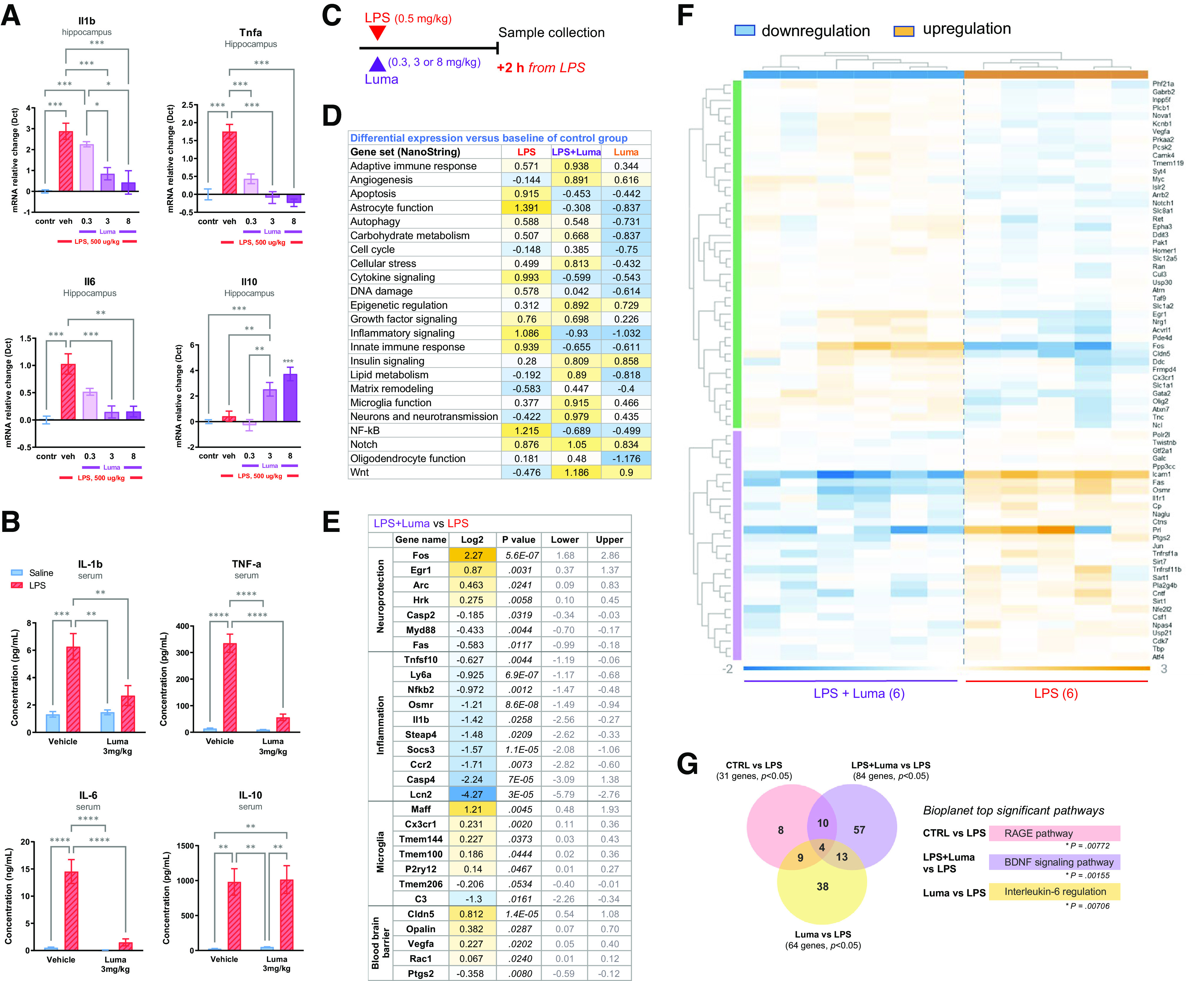
In LPS-induced inflammatory conditions, lumateperone conferred anti-inflammatory activity. The LPS dose was 500 µg/kg subcutaneously, and lumateperone or vehicle were coinjected intraperitoneally, immediately thereafter. Samples were collected 2 h after these injections, and mRNA was analyzed by qRT-PCR or NanoString Neuropath panel and serum by multiplex. ***A***, A dose–response was performed for lumateperone at 0.3, 3, and 8 mg/kg. Results show hippocampal mRNA levels of *Il1b*, *Tnfa*, *Il6*, and *Il10* mRNA normalized to the control group (in blue) using the Dct method; *n* = 5–12 per group. ***B***, The ability of lumateperone to normalize inflammation levels was confirmed in serum. Protein levels from blood serum were analyzed by Multiplex MSD assay V-Plex technology and expressed in pg/ml for IL-1β, TNF-α, and IL-10, and in ng/ml for IL-6. ***C***, The same study design was followed for NanoString analyses. ***D***, Directed global significance scores were calculated by nSolver software, which measures the extent to which a given gene set is upregulated or downregulated relative to a given covariate; the control group was used as the reference in this experiment. ***E***, Top genes involved in microglia function, neuroprotection, and inflammation that were altered (log2, *p* value, lower value, upper value) in LPS+Luma versus LPS groups. ***F***, Hierarchical clustering heatmap of LPS+Luma versus LPS showing significant genes upregulated (in orange) or downregulated (in blue) in hippocampal tissue. ***G***, Venn diagrams revealed poor overlap of significantly altered gene expression changes (*p* ≤ 0.04999) when group comparisons were performed. Graphs are mean ± SEM; *n* = 3-7 per group. *p < 0.05, **p < 0.01, ***p < 0.001, ****p < 0.0001, one-way ANOVA followed by Bonferroni multiple comparison test. For NanoString analyses, see Extended Data Figure 1-1 for a list of housekeeping genes that were used for expression normalization. BDNF, brain-derived neurotrophic factor; Contr, Control; Dct, δ ct; Luma, lumateperone; RAGE, receptor for advanced glycation end products; tnfa, tumor necrosis factor-α; Veh, vehicle.

Specific cytokines are elevated in serum or plasma of patients with MDD and other psychiatric disorders (Zhang et al., 2016). Here, we measured the gene and protein expression of a subset of pro- and anti-inflammatory cytokines in male mouse brain in response to an inflammatory challenge using a single dose of LPS (500 µg/kg) to induce acute brain inflammation. Samples were collected 2 h after coinjections of LPS and lumateperone or vehicle. The ability of lumateperone to ameliorate LPS-induced changes in male mouse hippocampal mRNA levels of these cytokines was initially studied using three doses of lumateperone (0.3, 3, and 8 mg/kg, i.p.). These doses span lumateperone's effective dose range for modulation of antipsychotic-like and antidepressant-like activity in rodents (Snyder et al., 2015). As expected, LPS treatment significantly increased gene expression for pro-inflammatory cytokines in hippocampus but did not significantly alter that of the anti-inflammatory cytokine *Il10* relative to control mice, as determined by one-way ANOVA analyses (effect of LPS treatment on levels of *Il1b*: *F*_(4,40)_ = 16.48, *p* < 0.01; *Il6*: *F*_(4,43)_ = 11.57, *p* < 0.001; *Tnfa*: *F*_(4,43)_ = 24.51, *p* < 0.001; *Il10*: *F*_(4,41)_ = 13.15, *p* > 0.99; Fig. 1*A*). Results showed that, when administered at the same time as LPS, lumateperone dose-dependently lowered LPS-induced elevations of hippocampal mRNA levels of the pro-inflammatory genes *Il1b*, *Tnfa*, and *Il6* (*Il1b*: doses of 3 and 8 mg/kg, *p* < 0.001; *Il6*: dose of 3 mg/kg, *p* < 0.001, 8 mg/kg, *p* < 0.01; *Tnfa:* all doses, *p* < 0.001; Fig. 1*A*). In addition, lumateperone significantly increased hippocampal mRNA levels of the anti-inflammatory cytokine *Il10* at doses of 3 and 8 mg/kg compared with levels seen in animals receiving LPS alone (*post hoc* comparison of means: *p* = 0.004 and *p* < 0.001 compared with LPS at lumateperone doses of 3 and 8 mg/kg, respectively; Fig. 1*A*).

**Figure 2.**
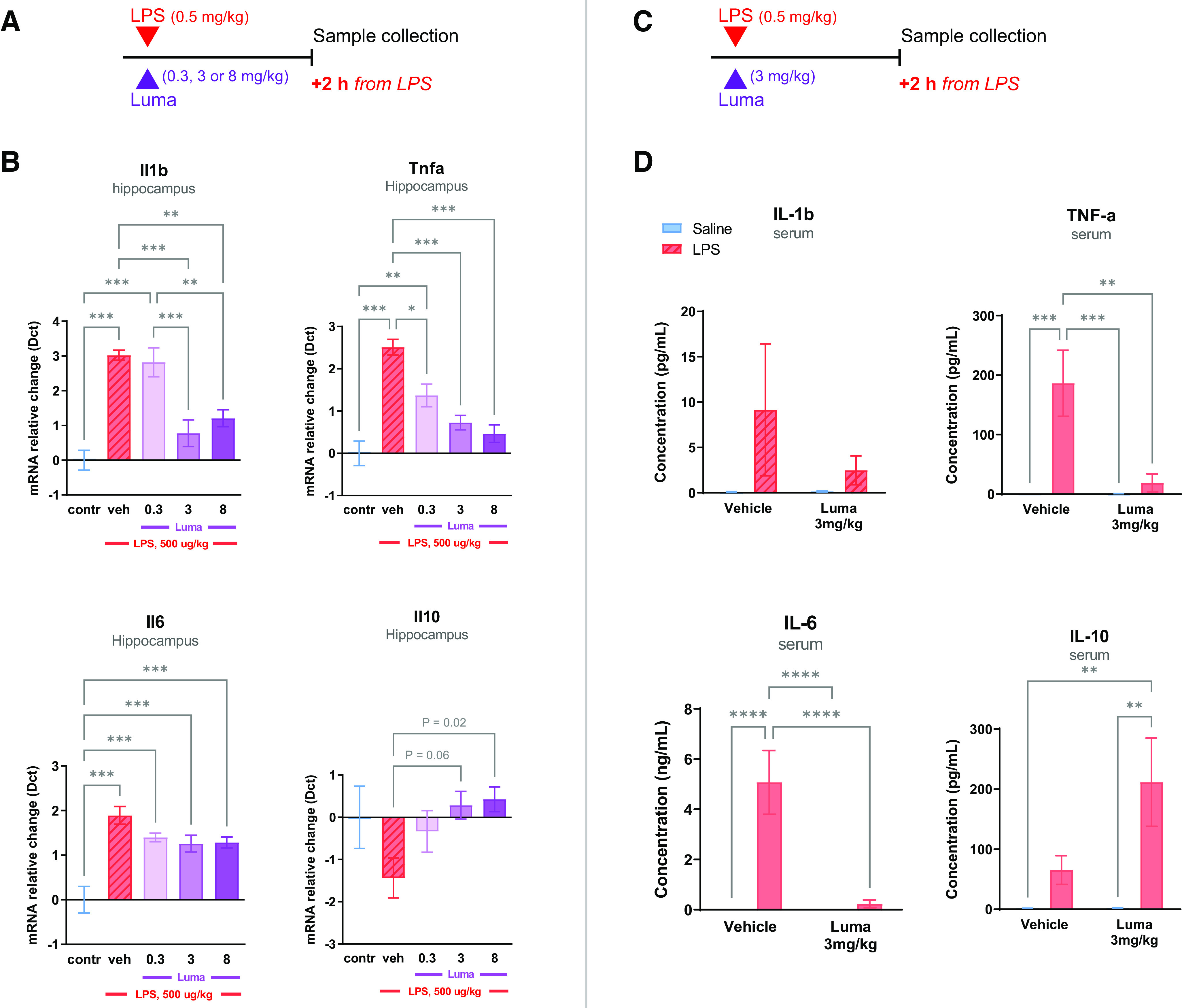
In female mice, LPS-induced inflammatory conditions were tempered by lumateperone. The doses used for LPS and lumateperone mirrored those of the study design for male mice. LPS (500 µg/kg subcutaneous) and lumateperone or vehicle were coinjected intraperitoneally, immediately thereafter. Samples were collected 2 h after these injections, and mRNA was analyzed by qRT-PCR or serum by multiplex. ***A–B***, A dose–response was performed for lumateperone at 0.3, 3, and 8 mg/kg. Results show hippocampal mRNA levels of *Il1b*, *Tnfa*, *Il6*, and *Il10* mRNA normalized to the control group (in blue) using the Dct method; *n* = 5–12 per group. ***C–D***, The ability of lumateperone to normalize inflammation levels was confirmed in serum in female mice injected with a dose of lumateperone of 3 mg/kg. Protein levels from blood serum were analyzed by Multiplex MSD assay V-Plex technology and expressed in pg/ml for IL-1β, TNF-α, and IL-10, and in ng/ml for IL-6. Graphs are mean ± SEM; *n* = 6-8 per group. *p < 0.05, **p < 0.01, ***p < 0.001, ****p < 0.0001, one-way ANOVA followed by Bonferroni multiple comparison test. contr, Control; Dct, δ ct; Luma, lumateperone; Tnfa, tumor necrosis factor-α; veh, vehicle.

A similar experiment was conducted in female mice and yielded similar results. LPS treatment significantly increased pro-inflammatory cytokines in the female hippocampus, but not Il10, relative to controls, as determined by one-way ANOVA analyses (effect of LPS on levels of *Il1b*: *F*_(4,33)_ = 16.58, *p* < 0.001; *Il6*: *F*_(4,35)_ = 13.00, *p* < 0.001; *Tnfa*: *F*_(4,34)_ = 18.18, *p* < 0.001; Fig. 2*A*,*B*). As in male mice, in female mice lumateperone dose-dependently lowered LPS-induced elevations of the pro-inflammatory genes *Il1b* and *Tnfa* (*Il1b*: doses of 3 and 8 mg/kg, *p* < 0.001 and *p* < 0.01, respectively; *Tnfa:* 0.3 mg/kg, *p* < 0.05, 3 and 8 mg/kg, *p* < 0.001; Fig. 2*B*). Lumateperone also significantly increased the anti-inflammatory cytokine *Il10* at 8 mg/kg compared with levels seen in animals receiving LPS alone (*p* < 0.05, Fig. 2*B*).

To determine whether lumateperone also reduces LPS-induced increases in pro-inflammatory cytokine protein levels in peripheral blood, a dose of 3 mg/kg lumateperone was selected for further analysis based on data from the above dose–response study in hippocampal tissue. An additional experimental group receiving an injection with lumateperone alone was included as an additional control. A pattern of results similar to that seen for gene expression changes in hippocampal tissue was obtained when examining protein levels of inflammatory biomarkers in serum. Two-way ANOVA revealed a significant effect of LPS, which elevated the protein levels of all biomarkers studied (Tukey multiple comparison vs control; IL-1β: *F*_(1,17)_ = 15.21, *p* < 0.0012; IL-6: *F*_(1,19)_ = 27.77, *p* < 0.0001; TNF-α: *F*_(1,20)_ = 69.12, *p* < 0.0001; IL-10: *F*_(1,16)_ = 38.24, *p* < 0.001; Fig. 1*B*). This trend was also observed in female mouse serum, with the exception of the IL-1β biomarker (Tukey multiple comparison vs control; IL-1β: *F*_(1,22)_ = 1.503, *p* = 0.23; IL-6: *F*_(1,27)_ = 16.00, *p* < 0.001; TNF-α: *F*_(1,28)_ = 12.48, *p* < 0.01; IL-10: *F*_(1,28)_ = 12.46, *p* < 0.01; Fig. 2*C*,*D*). As shown in Figure 1*B*, lumateperone treatment reduced circulating protein levels in serum of the pro-inflammatory cytokines IL-1β, TNF-α, and IL-6 compared with male mice treated with LPS alone (IL-1β: LPS = 6.267 pg/ml, LPS+Luma = 2.686 pg/ml, *p* = 0.0081; IL-6: LPS = 14.52 ng/ml, LPS+Luma = 1.494 ng/ml, *p* < 0.0001; TNF-α: LPS = 335.3 pg/ml, LPS+Luma = 56.33 pg/ml, *p* < 0.0001). Lumateperone also reduced circulating pro-inflammatory cytokines in female mouse serum compared with mice treated with LPS alone (IL-6: LPS = 5.075 ng/ml, LPS+Luma = 0.2377 ng/ml, *p* < 0.0001; TNF-α: LPS = 186.4 pg/ml, LPS+Luma = 18.69 pg/ml, *p* < 0.01; Fig. 2*D*). Previous work has shown that LPS, which is a cell wall component of Gram-negative bacteria, binds to Toll-like receptor 4 and activates nuclear factor κB (NFκB) signaling (Hoshino et al., 1999) as well as a mixed gene profile that is not strictly pro-inflammatory as it is known to also upregulate IL-10 signaling molecules in primary rodent microglia (Ledeboer et al., 2002; Das et al., 2017). Here too, in contrast to brain tissue, we report that LPS challenge increased IL-10 protein levels in male mouse serum (IL-10: control = 24.80 pg/ml, LPS = 980.4 pg/ml, LPS+Luma = 1014 pg/ml, Luma = 48.92 pg/ml; Fig. 1*B*). Two-way ANOVA did not reveal any drug effect (*F*_(1,16)_ = 0.03489, *p* = 0.8542) for IL-10 protein levels in serum, although lumateperone treatment by itself, without LPS, induced a significant IL-10 increase compared with control only (data not shown). The same is true for female mice; two-way ANOVA revealed a significant LPS challenge effect (IL-10: control = 1.725 pg/ml, LPS = 65.14 pg/ml, LPS+Luma = 211.7 pg/ml, Luma = 2.22 pg/ml; Fig. 2*D*). These data demonstrate that, compared with vehicle, lumateperone increased the protein levels of anti-inflammatory cytokine IL-10 while normalizing certain pro-inflammatory cytokines elevated by LPS in serum and brain of male and female mice.

To gain a deeper understanding of the transcriptional pathways and regulatory mechanisms altered by lumateperone in the context of elevated inflammation, we performed NanoString nCounter-based analysis following coinjection of LPS (500 µg/kg) and lumateperone (3 mg/kg) with sample collection 2 h after injection in male mice (Fig. 1*C*). The NanoString platform has been effectively used to quantitatively measure *in vivo* gene expression of target genes in several neuropathological mouse models. When coinjected with LPS, NanoString software analyses confirmed that lumateperone significantly decreased the expression of genes involved in inflammatory processes (Fig. 1*D*,*E*). Directed global significance scores, which in this experiment measured the extent to which a given gene set was upregulated or downregulated relative to the control group, showed that LPS+Luma treatment downregulated gene expression sets involved in cytokine signaling (LPS: 0.993, LPS+Luma: −0.599, Luma: −0.543), inflammatory signaling (LPS: 1.086, LPS+Luma: −0.93, Luma: −1.032), innate immune response (LPS: 0.939, LPS+Luma: −0.655, Luma: −0.611), and the NFκB pathway (LPS: 1.215, LPS+Luma: −0.689, Luma: −0.499) (Fig. 1*D*). Pathway analysis also documented an increase in genes associated with angiogenesis, epigenetic regulation, and Notch and Wnt pathways in groups injected with lumateperone. It is interesting to note that lumateperone alone often altered gene expression in these pathways to a comparable extent (i.e., scored similarly) as did combined treatment with LPS+Luma. NanoString software analyses also showed that compared with LPS alone, the LPS+Luma combination increased expression of markers of neuroprotection, such as *Fos*, *Egr1*, *Arc, Hrk, Myd88*, or *Fas*, while robustly decreasing expression of genes involved in inflammation, such as *Lcn2*, *Casp4*, *Ccr2*, *Socs3*, *Steap4, Il1b*, *Osmr*, *Ly6a, and Tnfsf10* (Fig. 1*E*). Microglia markers of homeostasis, including *Maff*, *Cx3cr1, Tmem144*, *Tmem100*, and *P2ry12*, were upregulated by lumateperone, while the gene acting as a regulator of the complement system, *C3*, was significantly downregulated, further supporting the potential protective properties of lumateperone in acute inflammatory conditions. *Cldn5* and *Opalin*, which are genes involved in BBB protection, were significantly upregulated by lumateperone. Other markers involved in BBB functionality were also altered in this group, such as *Vegfa*, *Rac1*, and *Ptgs2* (Fig. 1*E*).

Next, using ROSALIND Advanced Analysis Software and a filter set to *p* < 0.04999, we obtained a heatmap of cytokine-specific gene expression comparing LPS+Luma versus LPS alone (Fig. 1*F*). The data analyzed with ROSALIND confirmed that lumateperone significantly downregulated genes (shown in blue) that promote inflammation (e.g., *Osmr, Tnfrsf1a, Tnfrsf11b, Prl,* and *Il1r1*). Venn diagrams based on this analysis revealed some overlap of significantly altered gene expression changes (*p* ≤ 0.04999) when group comparisons were performed and indicated the following: receptor for advanced glycation end products pathway was significantly altered when comparing LPS versus control; BDNF signaling pathway was among the top most significant pathways altered in the group LPS+Luma versus LPS; and IL-6 regulation was also one of the top pathways when comparing Luma versus LPS (Fig. 1*G*). In summary, we discovered that lumateperone reversed acute inflammatory conditions by normalizing key pathways involved in inflammation in parallel with enhancing a gene signature indicative of tissue protection and repair.

### Lumateperone reduced preestablished LPS-induced proinflammatory cytokine mRNA levels in the hippocampus and reinforced BBB integrity

**Figure 3.**
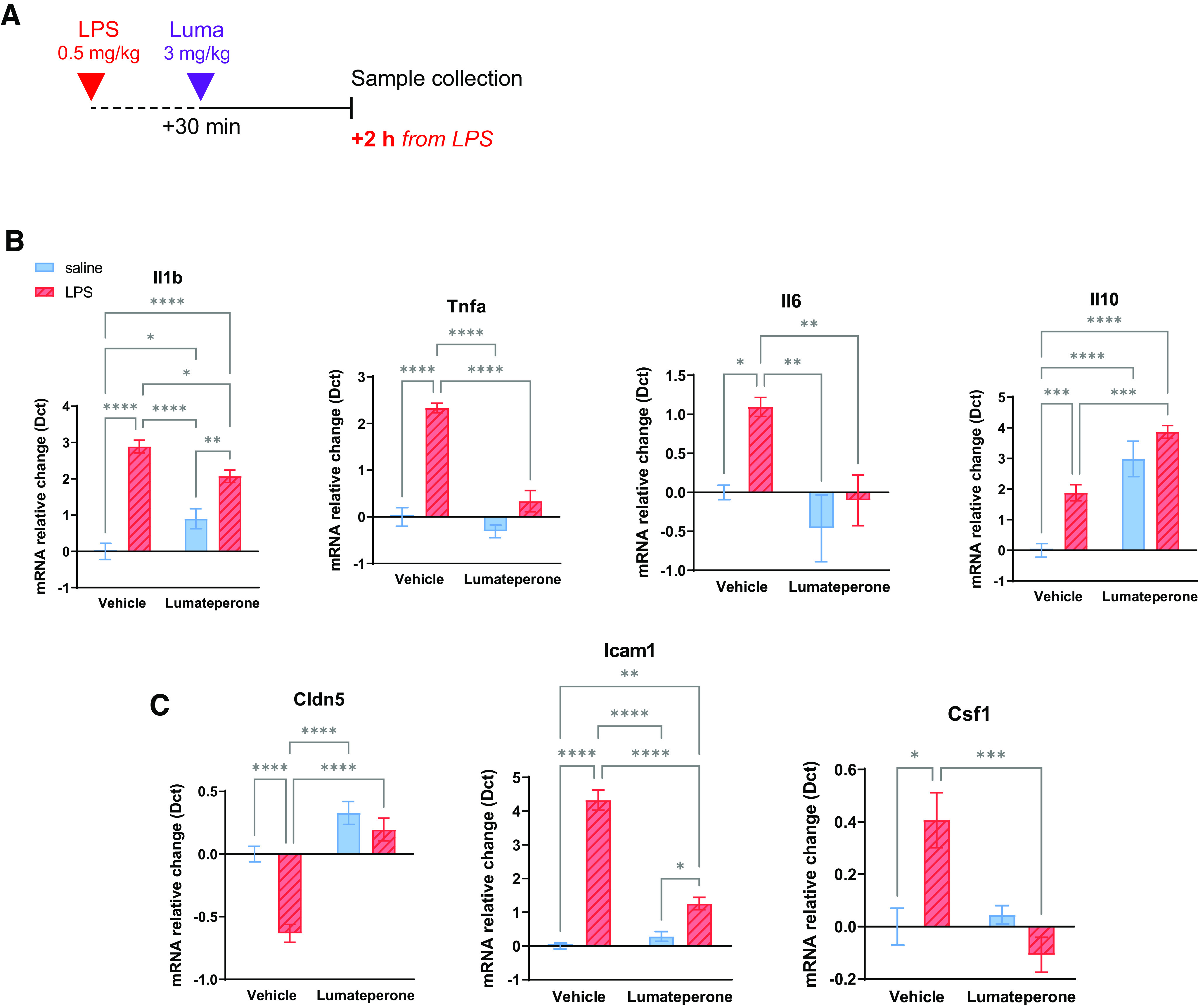
Delayed injection of lumateperone after LPS (30 min later) confirms anti-inflammatory activity in hippocampal tissue. ***A***, Adult mice first received a subcutaneous injection of either LPS (500 µg/kg diluted in 0.9% saline) or saline. Thirty minutes later, mice were injected intraperitoneally with either lumateperone (3 mg/kg) or its vehicle (5% DMSO, 5% Tween 20, 15% PEG 400, and 75% water). ***B***, Results show hippocampal mRNA levels of *Il1b*, *Tnfa*, *Il6*, *Il10*, and (***C***) *Icam1*, *Cldn5*, and *Csf1* normalized to the control group using the Dct method. Graphs are mean ± SEM; *n* = 4–9 per group. *p < 0.05, **p < 0.01, ***p < 0.001, ****p < 0.0001, compared with control group (two-way ANOVA followed by Tukey's multiple comparison test). The control group received vehicle+saline injections. Dct, δ ct; Luma, lumateperone; Tnfa, tumor necrosis factor-α.

Based on these findings, we sought to study whether a delayed administration of lumateperone would alter a preestablished state of elevated inflammation and thereby reestablish immune system homeostasis in male mice. Lumateperone (3 mg/kg, i.p.) or vehicle was administered 30 min after LPS injection, and samples were collected 1.5 h later (i.e., 2 h after LPS injection) (Fig. 3*A*). Again, LPS significantly increased hippocampal mRNA levels of *Il1b*, *Il6,* and *Tnfa* (two-way ANOVA, LPS effect: *Il1b*: *F*_(1,19)_ = 94.51, *p* < 0.0001; *Il6*: *F*_(1,20)_ = 8.008, *p* = 0.0104; *Tnfa*: *F*_(1,20)_ = 63.35, *p* < 0.0001), and the delayed injection of lumateperone reduced mRNA levels (*Il1b*: LPS = 2.891, LPS+Luma = 2.073, *p* = 0.0433; *Il6*: LPS = 1.096, LPS+Luma = –0.1023, *p* = 0.0078; *Tnfa*: LPS = 2.333, LPS+Luma = 0.3379, *p* < 0.0001; Fig. 3*B*). These results indicate that lumateperone exhibited similar effects when given as a coinjection with LPS or 30 min after the LPS injection and that the mRNA levels of *Il10* were elevated by lumateperone in the presence or absence of LPS. Two-way ANOVA analyses of *Il10* showed an effect of LPS (*F*_(1,20)_ = 21.31, *p* = 0.0002) and lumateperone (*F*_(1,20)_ = 69.02, *p* < 0.0001; LPS = 1.879; LPS+Luma = 3.869; Luma = 2.984), which confirms that lumateperone modulates hippocampal mRNA levels of this anti-inflammatory cytokine (Fig. 3*B*). In addition, we examined supplementary key markers revealed by our NanoString analyses. There was a significant interaction, treatment (LPS) effect by drug effect (lumateperone) for *Cldn5* and *Icam1*. Lumateperone decreased levels of *Icam1* (LPS = 4.327, LPS+Luma = 1.259, *p* < 0.0001) and coadministration of LPS+Luma increased *Cldn5* (LPS = −0.6332, LPS+Luma = 0.1962, *p* < 0.0001; Fig. 3*C*). Analyses of the levels of *Csf1* mRNA showed an interaction between drug and treatment and drug effect where lumateperone decreased *Csf1* relative to the LPS group (LPS = 0.4065, LPS+Luma = −0.1075, *p* = 0.0009; Fig. 3*C*). Collectively, these results indicated that transcriptional modulation of genes related to inflammation and tissue repair was initiated when lumateperone was administered at a delay following LPS-induced inflammation.

Systemic inflammation is associated with increased permeability of the BBB (Varatharaj and Galea, 2017); a potential factor underlying depression pathophysiology (Menard et al., 2017). Mice received a single injection of lumateperone (3 mg/kg, i.p.) at the same time (coinjection) or 30 min after (delayed) LPS injection. Forty-five minutes before sample collection, mice received NaFl injections (200 µl of 10% solution, i.p.; Table 2). Mice treated with LPS demonstrated significantly increased NaFl brain penetration, and this was significantly dampened in both the lumateperone coinjection group and in the lumateperone delayed injection group (control = normalized to 1, LPS = 1.406, control vs LPS: Tukey's multiple comparison test, *p* < 0.05; LPS+Luma = 0.863, LPS vs LPS+Luma: *p* < 0.01; LPS+Luma (delayed) = 0.652, LPS vs LPS+Luma (delayed): *p* < 0.001, all units in arbitrary units normalized to control; *F*_(3,21)_ = 11.49, *p* = 0.0001; Table 2). These data demonstrate that lumateperone administered in combination with LPS rescued the integrity of the BBB.

**Table 2.** Impact of lumateperone on cerebrum/serum NaFl fluorescence following LPS and restraint stress*^a^*

Condition (2 h)	Control	Experimental group	Experimental + Luma	Experimental + Luma (delayed)
LPS paradigm	1.000 ± 0.0957*	1.406 ± 0.0873	0.8632 ± 0.1273**	0.6515 ± 0.04580***
Restraint stress paradigm	1.000 ± 0.0311	1.132 ± 0.07244	0.7278 ± 0.1699^#^	—

*^a^*Lumateperone attenuated brain permeability to NaFl following LPS or restraint stress. For the LPS study cohort (*n* = 5-8), adult mice first received a subcutaneous injection of either LPS (500 µg/kg diluted in 0.9% saline) or saline. Then, mice were either coinjected or injected 30 min later intraperitoneally with lumateperone (3 mg/kg) or its vehicle (5% DMSO, 5% Tween 20, 15% PEG 400, and 75% water). For the restraint stress cohort (*n* = 4 or 5), after either lumateperone or vehicle treatment, mice were immediately placed in a restraint bag for a duration of 2 h while the control group went back into their cages. Forty-five minutes before sample collection, mice received NaFl injections. Ratios of cerebrum to serum NaFl fluorescence are represented as mean ± SEM. *p* < 0.05,

**p < 0.01,

***p < 0.001, compared with LPS group (one-way ANOVA followed by Tukey's multiple comparison test).

^#^*p* < 0.05 compared with stress group (unpaired *t* test). The control group received vehicle+saline injections. Luma, lumateperone.

### Lumateperone attenuated stress-induced inflammation and BBB permeability

**Figure 4.**
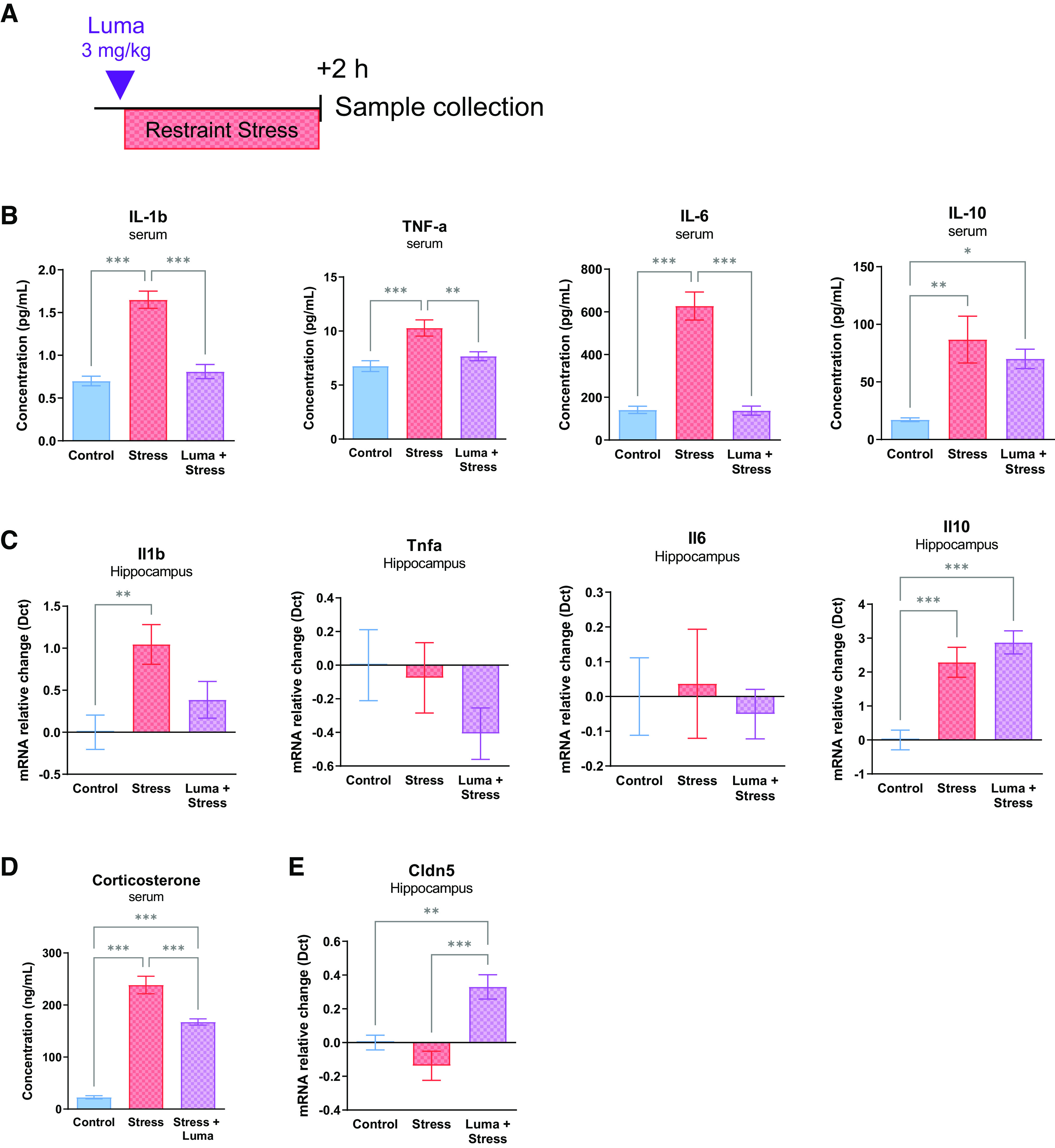
Lumateperone normalized markers of inflammation that were altered after 2 h of acute restraint stress. Adult mice were injected intraperitoneally with either lumateperone (3 mg/kg) or its vehicle (5% DMSO, 5% Tween 20, 15% PEG 400, and 75% water). For the groups receiving restraint stress, after treatment, mice were immediately placed in a restraint bag for a duration of 2 h while the control group went back into their cages. ***A***, Experimental design. ***B***, Results show blood serum protein levels of IL-1β, TNF-α, IL-6, and IL-10 normalized to control group, using Multiplex MSD V-plex technology where results are expressed in pg/ml. ***C***, Corresponding hippocampal levels of *Il1b*, *Tnfa*, *Il6*, and *Il10* mRNA normalized to control group (nonstressed, vehicle) using the Dct method. ***D***, Corticosterone was measured in serum with an ELISA. ***E***, *Cldn5* hippocampal mRNA levels. Graphs are mean ± SEM; *n* = 11–13 per group. *p < 0.05, **p < 0.01, ***p < 0.001, compared with control group (vehicle, nonstressed) using one-way ANOVA followed by Bonferroni multiple comparison test. Dct, δ ct; Luma, lumateperone; TNF-a, tumor necrosis factor alpha.

To determine whether lumateperone could normalize brain pathologic inflammation induced by an acute stressor, we used restraint stress which is a stressor known to evoke increases in inflammation (Buynitsky and Mostofsky, 2009). Mice received a single injection of lumateperone (3 mg/kg, i.p.) or vehicle immediately before being placed in a rodent restraint bag for 2 h; control mice received vehicle treatment and were returned to their home cage before sample collection (Fig. 4*A*). We demonstrated that acute restraint stress resulted in significant elevations in serum IL-1β, IL-6, and TNF-α levels while each of these proteins was significantly reduced to control levels in mice receiving lumateperone (IL-1β: Stress = 1.650, Stress+Luma = 0.8100, Bonferroni's multiple comparisons test, *p* < 0.001; IL-6: Stress = 627.4, Stress+Luma = 137.7, *p* < 0.001; TNF-α: Stress = 10.28, Stress+Luma = 7.669, *p* = 0.007, compared with controls. IL-1β: control = 0.700; IL-6: control = 141.3; TNF-α: control = 6.755, all values expressed in pg/ml, Fig. 4*B*).

In the hippocampus of the same mice, acute restraint stress led to an increase in mRNA levels for *Il1b* (control = normalized to zero, Stress = 1.045, *p* = 0.007), which were not significantly decreased after lumateperone treatment (Stress+Luma = 0.3858; Fig. 4*C*). At this time point, *Tnfa* and *Il6* mRNA levels were not altered by acute restraint stress. Interestingly, IL-10 serum protein and hippocampal mRNA levels were both increased by lumateperone compared with controls (IL-10 protein levels: control = 17.18 pg/ml, Stress+Luma = 70.03 pg/ml, Bonferroni's multiple comparisons test, *p* = 0.001; *Il10* mRNA levels: control = normalized to zero, Stress+Luma = 2.874, *p* < 0.001; Fig. 4*B*,*C*). In addition, corticosterone levels increased in blood serum of stressed mice and the elevated levels were significantly dampened by lumateperone (control: 22.65, Stress: 238.5, Stress+Luma: 167.3; all units in ng/ml; Stress vs Stress+Luma: *p* < 0.001; *F*_(2,27)_ = 124.2, *p* < 0.001; Fig. 4*D*). Here again, we confirmed that *Cldn5* transcripts were significantly elevated by lumateperone in stressed animals (Stress: −0.1376, Stress+Luma: 0.3300; Stress vs Stress+Luma: *p* < 0.001; *F*_(2,36)_ = 11.44, *p* < 0.001; Fig. 4*E*).

In a separate cohort, acute restraint stress did not significantly increase NaFl brain penetration (Control = normalized to 1, Stress = 1.132). However, lumateperone alone did significantly decrease NaFl brain penetration in the stress + lumateperone cohort compared with the stress cohort (Stress+Luma = 0.7278; Stress vs Stress+Luma: unpaired *t* test, *p* < 0.05; *t*_(7)_ = 2.373; Table 2).

### Lumateperone acted on rat microglia isolated from hippocampus after LPS-induced inflammation

**Figure 5.**
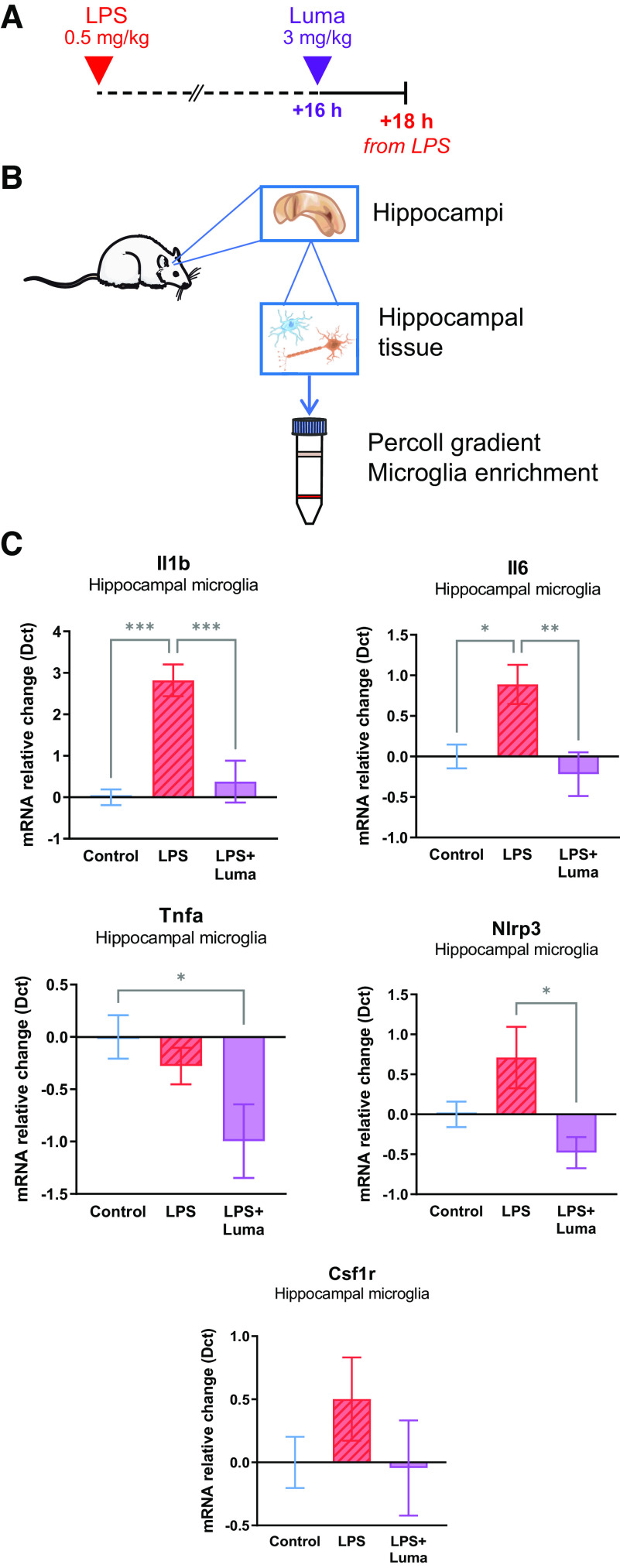
Lumateperone altered hippocampal microglia under inflammatory conditions. Lumateperone normalized inflammatory markers in microglia isolated from rat hippocampus 16 h after treatment with LPS. ***A***, Adult rats received a subcutaneous injection of either LPS (500 µg/kg diluted in 0.9% saline) or vehicle. Sixteen hours later, rats were injected intraperitoneally with either lumateperone (3 mg/kg) or its vehicle (5% DMSO, 5% Tween 20, 15% PEG 400, and 75% water). ***B***, Hippocampi were dissected and enriched for microglia via Percoll gradient. ***C***, Results show hippocampal microglia levels of *Il1b*, *Tnfa*, *Il6*, *Nlrp3*, and *Csf1r* mRNA normalized to control group using the Dct method. Graphs are presented as mean ± SEM; *n* = 8–12 per group; 5 cohorts of rats were pooled together. *p < 0.05, **p < 0.01, ***p < 0.001, compared with control group (two-way ANOVA followed by Tukey's multiple comparison test). Representative images in ***B*** were obtained via Servier Medical Art (2022) by Servier (https://smart.servier.com) under a Creative Commons Attribution 3.0 Unported License (https://creativecommons.org/licenses/by/3.0). Dct, δ ct; Luma, lumateperone; Tnfa, tumor necrosis factor-α.

Our purpose here was to investigate whether microglia could be involved in the suppression of LPS-induced inflammation mediated by lumateperone administration based on the associations revealed by gene ontology analyses. Microglia, the resident immune cells of the brain, have emerged as one likely effector for initiating and resolving neuroinflammation in a wide range of conditions and disorders (O'Brien et al., 2020; Woodburn et al., 2021a). Specifically, we examined the effect of lumateperone on *in vivo* inflammatory activity in hippocampal microglia and examined a time frame when inflammation could be detected in enriched preparations of rat brain microglia. Exploratory experiments revealed that in microglia-enriched fractions in brain homogenates, inflammation was returned close to background levels at 26 h after a dose of LPS (data not shown); thus, we selected an earlier time point of 18 h after the LPS injection to assess potential changes indicative of inflammation. Rats were pretreated with the same dose of LPS used for biochemical and RNA-based experiments (500 µg/kg) and received either lumateperone or vehicle injection 16 h later (for experimental design details, see Fig. 5*A*).

Hippocampus on both sides of the brain were collected 2 h later (18 h from LPS injection) and microglia were rapidly isolated in an enriched fraction (Fig. 5*B*). RNA was extracted from the resulting, reconstituted cell pellet and qRT-PCR confirmed that LPS treatment led to significant increases in *Il1b* and *Il6* gene expression in isolated hippocampal microglia; these increases were significantly suppressed by lumateperone administration (*Il1b*: control = normalized to zero, LPS = 2.819, LPS+Luma = 0.3773, one-way ANOVA *F*_(2,17)_ = 15.05, *p* < 0.001; *Il6*: control = normalized to zero, LPS = 0.8882, LPS+Luma = −0.2186, one-way ANOVA *F*_(2,20)_ = 6.622, *p* = 0.006; Fig. 5*C*). For *Nlrp3*, lumateperone significantly decreased the gene expression level compared with LPS alone (control = normalized to zero, LPS = 0.7106, LPS+Luma = –0.4790; one-way ANOVA, *F*_(2,26)_ = 4.302, *p* = 0.02; Fig. 5*C*). At this time point, however, *Tnfa* gene expression did not differ from controls, most likely reflecting difference in response time course in isolated microglia compared with that observed in whole tissue. Nevertheless, lumateperone administration led to decreased *Tnfa* mRNA levels in isolated microglia compared with microglia isolated from either control rats or LPS-treated rats (*Tnfa*: control = normalized to zero, LPS = −0.2781, LPS+Luma = −0.9956, one-way ANOVA *F*_(2,20)_ = 3.868, *p* = 0.04). LPS showed a trend toward increasing *Csf1r* mRNA levels in microglia while lumateperone tended to reduce this response (LPS = 0.5022, LPS+Luma = −0.04456; one-way ANOVA *F*_(2,29)_ = 0.9725, *p* = 0.39; Fig. 5*C*). Although not statistically significant, this trend paralleled the observed effects of lumateperone treatment in whole tissue. In summary, the data suggest that lumateperone suppressed LPS induction of a subset of proinflammatory genes expressed in isolated hippocampal microglia.

### Lumateperone normalized acute LPS-induced anhedonia

**Figure 6.**
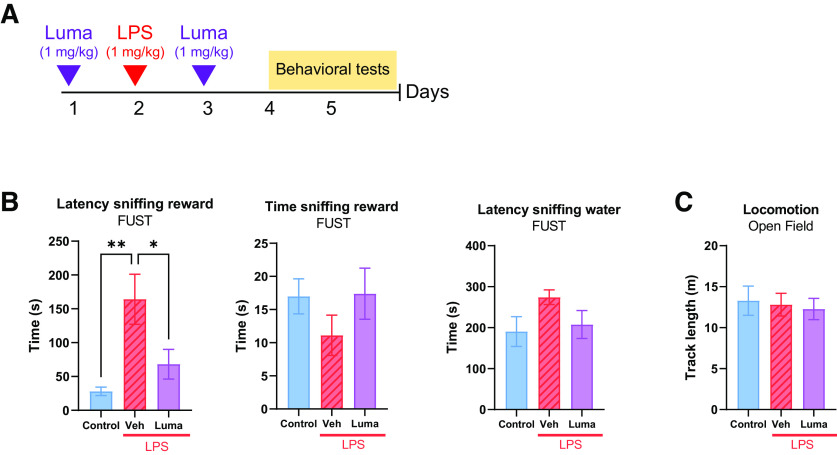
Lumateperone rescued transient anhedonic-like behavior and conferred anxiolytic properties to naive rats. ***A***, Lumateperone (1 mg/kg, intraperitoneal) was administered 24 h before and 24 h after a 1 mg/kg (subcutaneous) dose of LPS. ***B***, Anhedonia was assessed using FUST and measuring the latency to sniff the reward combined with time sniffing the reward. Latency sniffing water was used as a control. ***C***, Locomotion was evaluated in an open field. *p < 0.05. **p < 0.01. FUST, female urine sniffing test; Luma, lumateperone; Veh, vehicle.

Our next experiments determined whether lumateperone alters behavioral measures associated with anxiety/anhedonia that are known to be influenced by heightened inflammatory states. We administered LPS to induce a transient anhedonic state in rats and measured behaviors that rely on the reward system by using female urine as a rewarding stimulus to study whether lumateperone could rescue transient LPS-induced deficits. In a pilot study, a dose–response curve was conducted with varying doses of LPS to select an optimal dose for inducing an anhedonic response in rats (not shown). Based on these studies, a subcutaneous dose of 1 mg/kg LPS was selected. Pretreatment lumateperone (1 mg/kg, i.p.) was injected followed by LPS (1 mg/kg; s.c.) injection 24 h later and post-treatment lumateperone (1 mg/kg, i.p.) injection 24 h thereafter (Fig. 6*A*). Results showed that, when exposed to the reward cue (female urine), the LPS-treated rats who had been administered lumateperone exhibited a decreased latency to sniff the urine-soaked cotton tip compared with the LPS group (latency to sniff reward, control: 28 s, vehicle+LPS: 164.18 s, Luma+LPS: 68.12 s; Fig. 6*B*) and overall, lumateperone-treated rats spent as long as control rats sniffing the reward cue during the 5 min test period. Importantly, rats did not significantly differ with respect to the time spent exploring the water-dipped cotton tip that served as a control test (latency to sniff water, control: 190.55 s, vehicle+LPS: 274.27 s, Luma+LPS: 207.6 s). Locomotor activity was also not affected regardless of the group (Fig. 6*C*).

### Lumateperone reduced basal levels of anxiety and stimulated mTORC1 signaling pathway in the PFC

Here, we analyzed basal levels of anxiety with two commonly used tests, NSFT and NIH, in the absence of LPS. It is well documented that rodents experience increased stress levels when placed in a novel environment (Ramaker and Dulawa, 2017). These two tests exploit this feature by measuring latency to feed in food-deprived rats (NSFT), or latency to receive a reward to which they have been habituated before the test (NIH). Similar to the previous study, rats were injected with lumateperone (1 mg/kg, i.p.) on days 1 and 3. Lumateperone reduced the latency to feed in the NSFT (control: 657.8 s, Luma: 507.9 s, Mann–Whitney *U* test, *p* = 0.0009, Table 3). In contrast, there was no effect on feeding itself as shown in the HCFT, which is a control used for NSFT. Likewise in the NIH, which measures anxiety in a slightly different setting and does not require food deprivation, lumateperone reduced latency to drink the reward (i.e., diluted condensed milk; control: 65.4 s, Luma: 30.5 s, Mann–Whitney *U* test, *p* = 0.0257, Table 3) when rats were placed in a stress-inducing novel, empty, and brightly lit cage. Here again, locomotion assessed in an open field did not reveal a significant effect for lumateperone between treatment groups (Table 3).

**Table 3.** Behavioral tests to measure anxiety in naive rats injected with lumateperone*^a^*

	Control	Luma	Statistical test	*p*
NSFT (s)	657.8 ± 60.4	507.9 ± 55.7	Mann–Whitney *U*	0.0009***
Control HCFT (g)	6.0 ± 0.5	6.0 ± 0.3	Unpaired *t* test	0.9897 (NS)
NIH, novel cage (s)	65.4 ± 16.4	30.5 ± 13.8	Mann–Whitney *U*	0.0257*
OFT (m)	17.2 ± 3.4	32.8 ± 10.2	Unpaired *t* test	0.8879 (NS)

*^a^*Two doses of lumateperone (1 mg/kg) were administered on different days to a cohort of naive rats to assess anxiety-like behavior. Anxiety was investigated with NSFT paired with its control, HCFT, and NIH was measured in an empty novel cage. Locomotion was assessed in an open field. Data are mean ± SEM; *n* = 5–14 per group.

*p < 0.05,

***p < 0.001, compared with control group. Luma, lumateperone; NS, not significant.

**Figure 7.**
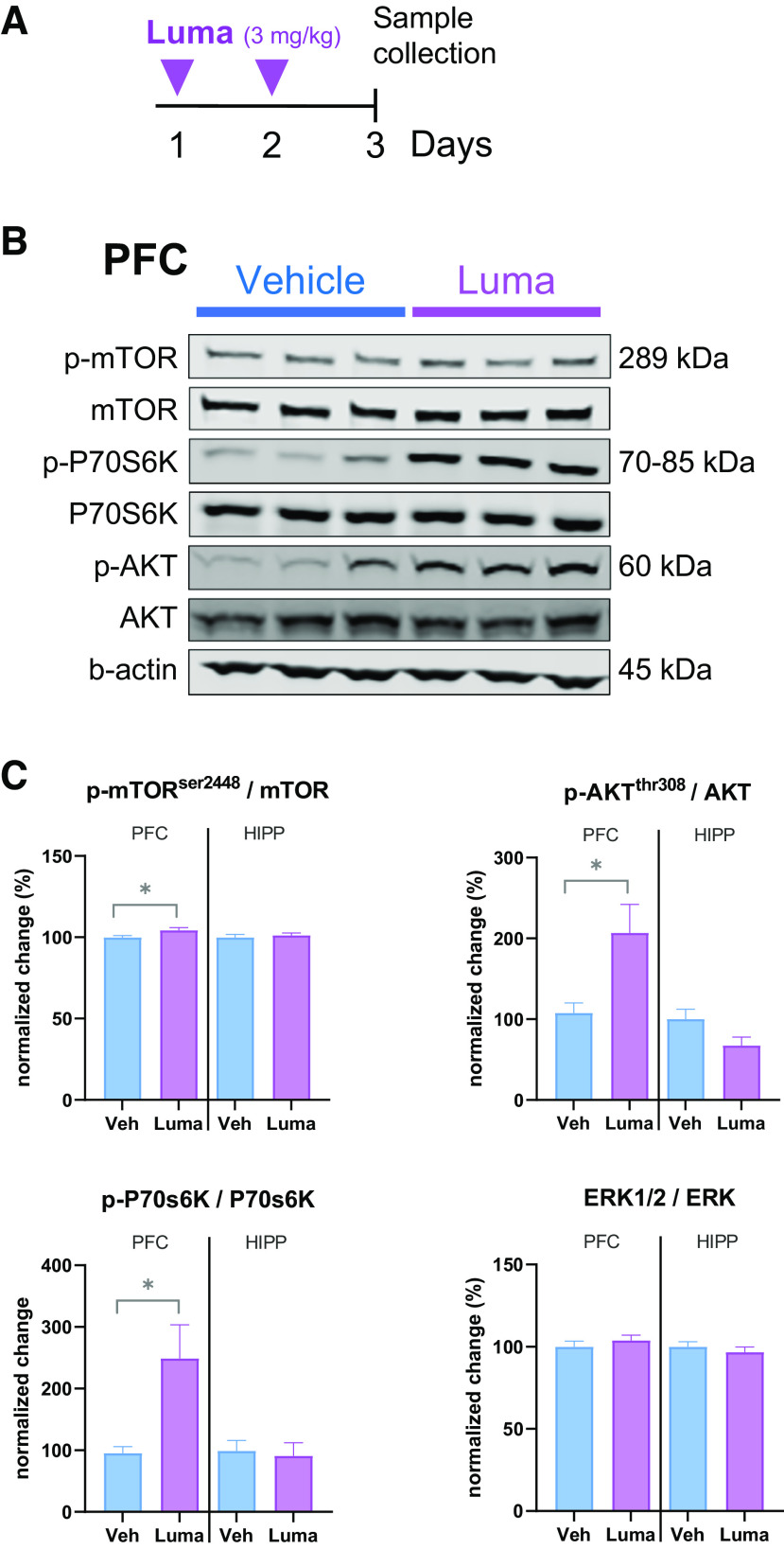
mTORC1 signaling is altered by lumateperone. ***A***, Male adult rats received either lumateperone (3 mg/kg, intraperitoneal) or vehicle on days 1 and 2. Sample collection happened on day 3. ***B***, Representative immunoblots for the two groups. ***C***, Quantitative results of phospho (p) protein immunoblots are based on total levels of protein for each particular kinase. Lumateperone differentially alters the phosphorylation of Akt, mTOR, and P70S6K in the PFC while changes are not observed at this time point in the hippocampus. Data were collected using Western blot analysis. Results are mean ± SEM; *n* = 19 or 20 per group. *p < 0.05 compared with the control vehicle group (two-tailed Student's *t* tests). Hipp, hippocampus; Luma, lumateperone; mTOR, mammalian target of rapamycin; PFC, prefrontal cortex; Veh, vehicle.

Next, we investigated whether some well-known mechanisms of action might be involved in these behavioral effects. Considering that some rapid-acting antidepressants increase upstream and downstream effectors of mTORC1 signaling in rodent PFC, we examined this pathway following two injections of lumateperone in rats. Results showed that 24 h after the second injection of lumateperone, there were increases of phospho-Akt (92.23%, *p* = 0.0115, Fig. 7), phospho-mTOR (4.33%, *p* = 0.0356, Fig. 7), and phospho-P70s6k (53.85%, *p* = 0.0111, Fig. 7) in the PFC. In addition to affecting synaptic plasticity, this pathway is also involved in the pathophysiology of stress-induced behavior. In summary, these results extend previous data (Snyder et al., 2015) confirming that lumateperone has the potential to reduce anhedonia and to decrease basal levels of anxiety in a stressful situation, while stimulating activity in the mTORC1 signaling pathway.

## Discussion

According to our findings in rodents, lumateperone ameliorates pathologic levels of inflammation in serum and hippocampal tissue, and especially in microglia. Following either an inflammogen or acute restraint stress, lumateperone was found to reduce key pro-inflammatory markers, such as IL-1β, IL-6, and TNF-α, when administered over a range of doses and time points. These pro-inflammatory biomarkers are known to be elevated in certain patients with psychiatric disorders (Maes et al., 1992; Hodes et al., 2014; Miller and Raison, 2016) and in human postmortem tissues, including PFC, from suicide victims (Lindqvist et al., 2009; Pandey et al., 2012).

Interestingly, we also found that lumateperone treatment reduced expression of the *Nlrp3* inflammasome, a large multiprotein complex involved in innate immunity (Iwata et al., 2016). Although the inflammasome has no baseline activity, once activated by stress (Iwata et al., 2016), infections, or other stimuli, it generates active forms of the inflammatory cytokines IL-1β and IL-18. In preclinical studies, *Nlrp3*-null mutant mice were resilient to the effects of stress on depression-like behavior (Iwata et al., 2016; Yue et al., 2017), and *Nlrp3* expression was increased in peripheral blood mononuclear cells from untreated patients with MDD (Alcocer-Gómez et al., 2014). Hence, targeting this pathway has been suggested as a promising approach for treating stress-related disorders and depression (Iwata et al., 2013; Franklin et al., 2018). Our results show that lumateperone treatment decreased *Nlrp3* transcript levels under conditions evoking pathologic inflammation, which may contribute, in part, to the antidepressant-like action of lumateperone (Snyder et al., 2015).

Stress and inflammation can compromise BBB integrity and functionality in many pathologic states (Menard et al., 2017). The BBB regulates ion and nutrient exchange between the brain and blood while protecting brain tissue from harmful agents. Malfunctioning BBB can result in chemical exposure and infections, and data suggest that the BBB may be compromised in persons with schizophrenia (Greene et al., 2018) and depression (Wu et al., 2022). Here, we observed an increased RNA copy number of hippocampal *Cldn5* in naive mice receiving lumateperone and confirmed these results in the brain of acutely stressed or LPS-treated mice. Claudins are small proteins (20-27 kDa) expressed in tight junctions between brain endothelial cells to maintain BBB integrity (Greene et al., 2019). In mice, *Cldn5* ablation enhanced BBB permeability and allowed infiltration of large proteins up to ∼69 kDa (e.g., IL-6) into mouse brain parenchyma and was associated with depressive-like behavior and behavioral impairments characteristic of schizophrenia and depression (Menard et al., 2017; Greene et al., 2018; Ma et al., 2018). Moreover, TNF-α/NFκB signaling increases BBB permeability by decreasing expression of Cldn5 (Dudek et al., 2020). Whereas chronic but not acute imipramine treatment rescued social avoidance and restored Cldn5 levels that were altered by social defeat stress (Menard et al., 2017), acute lumateperone treatment is efficacious in improving similar behavioral states. Our finding highlights another potential difference between classic antidepressant and lumateperone treatment. Since we observed reduced NaFl brain uptake and differences in anti-inflammatory cytokine expression between the CNS and the periphery, we suggest that Cldn5 may change early after lumateperone administration, resulting in the preservation of BBB integrity and limit of infiltration of large proteins (e.g., IL-6), infectious agents, and other potential pro-inflammatory stimuli that circulate in blood. Classical antidepressants have also been reported to modulate levels of ICAM-1, a cell adhesion molecule and a member of the immunoglobulin gene superfamily, which is involved in leukocyte brain infiltration and BBB hyperpermeability (Muller, 2019). Levels of ICAM-1 were shown to be increased in the orbitofrontal cortex of patients with depression (Miguel-Hidalgo et al., 2011). Our transcription profiling results coupled with the *in vivo* index of BBB functional recovery further support a crucial involvement of lumateperone in mediating preservation of BBB integrity. Since other genes regulated in endothelial cells involved in BBB function were also altered by lumateperone administration, it is reasonable to assume that the sequence of events may start with reinforcement of endothelial integrity. Alternately, increased monoaminergic/glutamatergic signaling may be responsible for mediating BBB enhanced protection via microglia released factors, particularly since we have reported that lumateperone acts on hippocampal microglia, which is an often overlooked component of the neurovascular unit (Kisler et al., 2021). Further work will be required to elucidate effects of these mechanisms on immune function in the brain.

Collectively, our data suggest that lumateperone's repertoire encompasses signaling networks involved in a variety of biological processes relevant to maintenance of BBB integrity and control of deleterious inflammatory states.

Increased BBB integrity activates microglia and leads to changes in microglia phenotype (Ronaldson and Davis, 2020). Accordingly, our NanoString analysis suggested that in acute inflammatory conditions, lumateperone significantly increased expression of genes related to microglia physiological functions and anti-inflammatory phenotype and decreased expression of microglia markers related to immune modulation. In the CNS, microglia are an important component of the local brain immune response. Here we found that enriched hippocampal microglia recapitulated the anti-inflammatory responses seen in whole-brain homogenates. One of the genes overexpressed in hippocampi from LPS-treated mice was *Csf1*. This gene encodes the ligand for the microglia receptor CSF1R, which is involved in maintaining microglia viability and immunologic surveillance (Bollinger and Wohleb, 2019); LPS-induced increases in *Csf1* expression were significantly reduced with lumateperone coadministration. Interestingly, a study reported increased *Csf1* mRNA in isolated microglia from mice submitted to chronic unpredictable stress and further, mice bearing a knockdown of neuron-derived *Csf1* failed to develop chronic unpredictable stress–induced anxiety- and depressive-like behaviors (Wohleb et al., 2018). Similarly, mice treated with a CSF1R antagonist (PLX5622) did not display monocyte recruitment to the brain or anxiety after social defeat (Weber et al., 2019). Furthermore, we found that lumateperone upregulated the anti-inflammatory cytokine IL-10, which could add to the repertoire of inflammation-resolving mechanisms following abnormal levels of stress and inflammation, possibly by influencing microglia function. Overall, our data show that the ability of lumateperone to alter microglial gene expression and to reduce *Csf1* gene expression following an inflammatory challenge may prevent activation of microglial function after exposure to proinflammatory stimuli.

Inflammatory cytokines can be released from microglia and influence synaptic plasticity (Khairova et al., 2009). Adaptive synaptic plasticity is a process by which neurons and circuits change their excitability and connectivity based on the strength, number, and density of synapses (Cholewinski et al., 2021). As neuronal circuits underlying mood regulation are known to be disrupted by traumatic or chronic stress (Duman et al., 2016; Cholewinski et al., 2021), synaptic connectivity has been particularly studied based on the discovery that stress causes atrophy of stress-vulnerable hippocampal neurons and pyramidal neurons in the medial PFC. Microglia, which are known to be involved in synaptic pruning during development, could also participate in atrophy and loss of spine synapses in the context of stress and depression (Woodburn et al., 2021b). Concomitant decreases in AMPARs, neurogenesis, BDNF-TRKB pathway, and mTORC1 signaling have been reported in both depressed patients (Jernigan et al., 2011; Kang et al., 2012) and animal models mimicking aspects of depressive disorders (Duman, 2018; N. Li et al., 2010, 2011; Zhou et al., 2014). These deficits were documented to be reversed quickly with administration of rapid-acting antidepressants, such as ketamine (Zhou et al., 2014; N. Li et al., 2010, 2011). In the current study, we demonstrate that acute administration of lumateperone stimulates PFC mTORC1 signaling pathway, as well as some important genes coding for neurotrophic factors, such as BDNF and VEGF, whose levels participate in neuronal protection and synaptic health. mTOR is a highly conserved serine/threonine kinase whose activity is stimulated by growth factors. Specifically, in the brain, mTORC1 multiprotein complex is involved in the regulation of synaptic plasticity, corticogenesis, and associated functions of neurons (Duman et al., 2016; Cholewinski et al., 2021). This signaling pathway has emerged as a surrogate target for new rapid-acting antidepressants. Importantly, here we found that the same lumateperone acute treatment regimen stimulating mTORC1 upstream and downstream effectors in the rat PFC also conferred anxiolytic effect and reversed a form of anhedonia, a hallmark feature of depression. In future research, we would need to measure other modalities related to reward-seeking measures, such as hedonic food intake, and include female subjects as well. Additional time points between 30 min to several weeks, as well as lumateperone chronic treatment, will be needed to fully characterize the full range of effects of this drug on long-term synaptic plasticity in the brain, as well as on behavioral outcomes in both sexes that will provide further evidence relevant to translational research.

Overall, stress and agents that elicit neuroinflammation are strongly implicated in the etiology of diverse brain disorders (Burgdorf et al., 2019; Schonhoff et al., 2020). Many infectious agents, including viruses, can trigger psychiatric pathology in part by elevating pro-inflammatory cytokines (Jelinek et al., 2012; Prossin et al., 2015; Burgdorf et al., 2019). We propose that therapeutics with anti-inflammatory benefit, such as lumateperone, may provide further advantage in normalizing aberrant neuroinflammatory events and mitigating their impact on brain dysfunction, particularly with reference to maintenance of BBB integrity.

In conclusion, mood disorders, including MDD and bipolar disorders, afflict a substantial proportion of the population, representing a huge socioeconomic burden. An urgent need exists for enhanced understanding of new therapeutic targets to address these disorders, particularly for the large percentage of patients with depression that is resistant to current treatments and for whom effective treatments are slow in providing benefit. Amelioration of aberrant and potentially pathologic inflammation is a rapidly emerging area of interest for new treatments for depression and psychiatric disorders. Our study demonstrates anti-inflammatory actions of lumateperone following direct immune system challenge and psychological stress. Mechanistically, the effects of lumateperone appeared to be mediated through immunologic pathways involved in mood regulation (e.g., NLRP3 inflammasome complex) involving, in part, microglia activation and regulation of proinflammatory cytokines, such as IL-6, IL-1β, and TNF-α. Lumateperone treatment also increased expression of the mTORC1 signaling pathway, the inflammation-resolving cytokine IL-10, and the tight junction protein CLDN5 which is involved in maintaining BBB integrity. We also report that lumateperone limited ICAM1 expression following exposure to inflammatory stimuli, potentially helping to curb peripheral damaging inflammatory conditions that secondarily impact the brain. Hence, lumateperone's mechanistic repertoire encompasses signaling networks involved in a variety of biological processes relevant to maintenance of brain tissue integrity. Considering the high comorbidity of mood disorders and inflammation in other diseases, including type 2 diabetes, cancer, stroke, COVID-19, and rheumatoid arthritis, these results provide a basis for further exploration of the clinical benefits of lumateperone in the treatment of a wide range of immune pathologies.

10.1523/JNEUROSCI.0984-22.2022.f1-1 Figure 1-1 Housekeeping genes selected for gene expression normalization in NanoString analyses. Download Figure 1-1, DOCX file.
