# Supplementary material for: Lumateperone Normalizes Pathological Levels of Acute Inflammation through Important Pathways Known to Be Involved in Mood Regulation
Source: J Neurosci. 2023 Feb 1;43(5):863–77. doi: 10.1523/JNEUROSCI.0984-22.2022 (PMC9899083; doi:10.1523/JNEUROSCI.0984-22.2022)
Supplement: Figure 1-1 — Housekeeping genes selected for gene expression normalization in NanoString analyses. Download Figure 1-1, DOCX file. [file ns-JN-RM-0984-22-s01.docx]

Figure 1-1. Housekeeping genes selected for gene expression normalization in NanoString analyses

| **Gene Name** | **Gene** |
| --- | --- |
| Aars-mRNA | NM_146217.4:716 |
| Asb10-mRNA | NM_080444.4:1884 |
| Ccdc127-mRNA | NM_024201.3:204 |
| Cnot10-mRNA | NM_153585.5:714 |
| Csnk2a2-mRNA | NM_009974.3:1066 |
| Fam104a-mRNA | NM_138598.5:1120 |
| Gusb-mRNA | NM_010368.1:1735 |
| Lars-mRNA | NM_134137.2:945 |
| Mto1-mRNA | NM_026658.2:1058 |
| Supt7l-mRNA | NM_028150.1:180 |
| Tada2b-mRNA | NM_001170454.1:3224 |
| Tbp-mRNA | NM_013684.3:70 |
| Xpnpep1-mRNA | NM_133216.3:1826 |
